# Machine Learning Model of ResNet50-Ensemble Voting for Malignant–Benign Small Pulmonary Nodule Classification on Computed Tomography Images

**DOI:** 10.3390/cancers15225417

**Published:** 2023-11-15

**Authors:** Weiming Li, Siqi Yu, Runhuang Yang, Yixing Tian, Tianyu Zhu, Haotian Liu, Danyang Jiao, Feng Zhang, Xiangtong Liu, Lixin Tao, Yan Gao, Qiang Li, Jingbo Zhang, Xiuhua Guo

**Affiliations:** 1 Department of Epidemiology and Health Statistics, School of Public Health, Capital Medical University, Beijing 100069, China; lwming@ccmu.edu.cn (W.L.); ysq@mail.ccmu.edu.cn (S.Y.); yrh@mail.ccmu.edu.cn (R.Y.); tianyixing@mail.ccmu.edu.cn (Y.T.); zhutianyu@mail.ccmu.edu.cn (T.Z.); lht20020108@mail.ccmu.edu.cn (H.L.); jiaodanyang@mail.ccmu.edu.cn (D.J.); zhangfeng@ccmu.edu.cn (F.Z.); lxiangtong@ccmu.edu.cn (X.L.); taolixin@ccmu.edu.cn (L.T.); 2 Beijing Municipal Key Laboratory of Clinical Epidemiology, Capital Medical University, Beijing 100069, China; 3 Department of Nuclear Medicine, Xuanwu Hospital Capital Medical University, Beijing 100053, China; wsggy518@sina.com; 4 Beijing Physical Examination Center, Beijing 100050, China; lifrancis126@163.com (Q.L.); 13910625118@139.com (J.Z.)

**Keywords:** ResNet50, ensemble voting, XGBoost, small pulmonary nodules, pulmonary cancer

## Abstract

**Simple Summary:**

Machine learning methods have shown promise in accurately identifying small lung nodules. However, further exploration is needed to fully harness the potential of machine learning in distinguishing between benign and malignant nodules. This study aimed to develop and evaluate a ResNet50-Ensemble Voting model for detecting the nature (benign or malignant) of small pulmonary nodules (less than 20 mm) based on CT images. This study involved 834 CT imaging data from 396 patients with small pulmonary nodules. CT image features were extracted using ResNet50 and VGG16 algorithms, and classification was performed using XGBoost, SVM, and Ensemble Voting techniques, incorporating ten different combinations of machine learning classifiers. Among the models tested, the ResNet50-Ensemble Voting algorithm demonstrated the highest performance in the test set, achieving an accuracy of 0.943 (0.938, 0.948), with sensitivity and specificity values of 0.964 and 0.911, respectively. The implementation of machine learning models, particularly the ResNet50-Ensemble Voting approach, showed excellent performance in accurately identifying benign and malignant small pulmonary nodules (less than 20 mm) from diverse sources. These models have the potential to assist doctors in accurately diagnosing the nature of early-stage lung nodules in clinical practice.

**Abstract:**

Background: The early detection of benign and malignant lung tumors enabled patients to diagnose lesions and implement appropriate health measures earlier, dramatically improving lung cancer patients’ quality of living. Machine learning methods performed admirably when recognizing small benign and malignant lung nodules. However, exploration and investigation are required to fully leverage the potential of machine learning in distinguishing between benign and malignant small lung nodules. Objective: The aim of this study was to develop and evaluate the ResNet50-Ensemble Voting model for detecting the benign and malignant nature of small pulmonary nodules (<20 mm) based on CT images. Methods: In this study, 834 CT imaging data from 396 patients with small pulmonary nodules were gathered and randomly assigned to the training and validation sets in an 8:2 ratio. ResNet50 and VGG16 algorithms were utilized to extract CT image features, followed by XGBoost, SVM, and Ensemble Voting techniques for classification, for a total of ten different classes of machine learning combinatorial classifiers. Indicators such as accuracy, sensitivity, and specificity were used to assess the models. The collected features are also shown to investigate the contrasts between them. Results: The algorithm we presented, ResNet50-Ensemble Voting, performed best in the test set, with an accuracy of 0.943 (0.938, 0.948) and sensitivity and specificity of 0.964 and 0.911, respectively. VGG16-Ensemble Voting had an accuracy of 0.887 (0.880, 0.894), with a sensitivity and specificity of 0.952 and 0.784, respectively. Conclusion: Machine learning models that were implemented and integrated ResNet50-Ensemble Voting performed exceptionally well in identifying benign and malignant small pulmonary nodules (<20 mm) from various sites, which might help doctors in accurately diagnosing the nature of early-stage lung nodules in clinical practice.

## 1. Introduction

Currently, lung cancer remains one of the leading causes of cancer-related mortality worldwide. It was estimated that there would be about 1.8 million deaths from lung cancer in 2020, accounting for 18% of all cancer deaths [1]. Screening for benign and malignant lung nodules in the early stages of lung cancer could increase the 5-year survival rate from 16.1% to 19.7% [2]. Therefore, the early detection and accurate classification of pulmonary nodules are crucial for improving patient prognosis. The gold standard cannot be relied on to distinguish the benign or malignant nature of small lung nodules in the early stages of lung cancer, because the nodules are too small to acquire pathologic evidence of the lung nodules [3]. Transbronchial biopsies of isolated pulmonary nodules (SPNs) smaller than 20 mm are typically performed under fluoroscopic guidance, but there is great variability in the availability of pathologic tissue [4]. As a result of its high resolution and non-invasive nature, computed tomography (CT) imaging has emerged as a significant tool in the identification and therapy of lung nodules [5]. A study demonstrated that annual lung cancer screening using CT imaging reduced lung cancer mortality by 20% [6]. However, radiologists continued to struggle with reliably discriminating between malignant and benign, small pulmonary nodules based only on CT imaging. Computer-assisted diagnostic tools had the potential to improve the detection and screening of benign and malignant lung nodules [7]. Kaliyugarasan applied a new extension of the fastai deep learning framework to a 3D medical imaging task and combined it with the MONAI deep learning library to achieve a final classification accuracy of 92.4% [8]. Zhao constructed a hybrid CNN of LeNet and AlexNet to distinguish benign and malignant lung nodules using CT images, and the accuracy and area under the curve reached 0.822 and 0.877, respectively, obtaining better results [9]. Keyan proposed an MSM-ViT model aiming to achieve promising performance in lung nodule classification, solving the problems of the poor generalization of ViT structure and the difficulty in extracting multi-scale features, and the best accuracy of 94.04% was obtained [10]. Mkindu proposed 3D-CNN and squeeze-and-excitation networks, with the joint algorithm yielding the highest detection sensitivity of 98.65% [11]. Hassan proposed an automated computer-aided diagnosis (CAD) scheme for lung nodule detection based on the Vision Transformer architecture and Bayesian optimization, obtaining 98.39% of the highest detection sensitivity with a significant reduction in network parameters [12]. However, the preceding research focused mostly on regular-sized lung nodules and did not investigate the model’s capabilities in small lung nodules (<20 mm).

There are studies that have used Bayesian penalized likelihood reconstruction [13] or maximum-intensity projection [14] to enhance the representation of small lung nodules on CT images to improve clinicians’ identification and the diagnosis of early-stage lung cancer. However, the diagnosis has not been made accurately to a certain extent. Kum used the modified AlexNET algorithm for the diagnosis of the nature of lung nodules smaller than 20 mm with an AUC value of 0.82, exploring the application of this method for the diagnosis of early lung nodules [15]. Mei introduced the Otsu thresholding algorithm to preprocess the data and filter the interfering information, obtaining nodule features, and parallel radiomics was added to the 3D convolutional neural network, reaching an AUC of 0.90 [16]. However, the capability to diagnose lung nodules in early stages remained insufficient, and further exploration and enhancement were desirable. Liu achieved superior outcomes by combining CT radiomics with machine learning to predict the invasiveness of small nodules [17]. Classical machine learning classification algorithms such as XGBoost [18] and SVM (Support Vector Machine) [19] have achieved better performance in diagnosing the nature of lung nodules. However, the diagnosis of small lung nodules in the early stage needed to be further explored. Furthermore, machine learning classification models based on feature extraction were further developed and explored. The Local Mesh Peak-Valley Edge Pattern (LMePVEP) technique for splicing-based feature extraction based on dynamic thresholding could improve the classification accuracy by up to 12.56% [20]. However, the accuracy of this method for the diagnosis of the nature of lung nodules still needed to be promoted. The ResNet18 scheme combined with different classifiers helped to achieve better accuracy, such as the SoftMax (95.2%) classifier and Decision Tree Classifier (99%), in lung disease recognition [21]. Therefore, the concept of extracting features based on deep learning and combining different classifiers for disease classification model construction was proven to be feasible.

In this study, we utilized a combination of deep learning feature extraction and different classifiers to construct a fusion model to explore and improve the diagnostic capability of the benign and malignant nature of lung nodules (<20 mm) in the early stage.

## 2. Materials and Methods

### 2.1. Data Source

From 2015 to 2019, 396 individuals were recruited for this study from four hospitals and two open access databases, and informed consent was obtained. All patients’ lung CT images were obtained in DICOM format, with a total of 934 layers involving pulmonary nodules. We adopted a questionnaire to collect clinician diagnoses and basic demographic information after analyzing patients’ medical records and admission data. A checklist of the subjects and the images is shown in Table 1.

It was further analyzed whether there was a difference in age and gender between patients with benign or malignant lung nodules. However, the results showed that no statistical difference was found between the two, which is shown in Table 2.

#### 2.1.1. Inclusion/Exclusion Criteria

Inclusion criteria:

- The subjects of this study should be adults (age ≥ 18 years);
- In order to ensure the integrity of the information in the lung nodule images, the number of CT images containing nodules should not be less than 2 per patient;
- Clear physician’s diagnostic report was available;
- Small pulmonary nodules less than 20 mm in size for which a definitive pathologic diagnosis cannot be made.

Exclusion criteria:

- Patients treated with chemo-radiotherapy or surgery;
- Images of nodules that were difficult to segment;
- The size of the lung nodule was above 20 mm.

#### 2.1.2. Diagnostic Criteria

This study utilized the gold standard for lung nodules with a clear pathologic diagnosis of the nature of small lung nodules, and in instances when a pathologic diagnosis could not be obtained due to the small size of the lung nodule, the diagnostic report based on the clinician’s a priori knowledge prevailed. The Chinese Expert Consensus on the Diagnosis and Treatment of Lung Nodules (2018 edition) contains detailed diagnostic criteria for lung nodules.

### 2.2. Research Design Process

**Figure 1:**
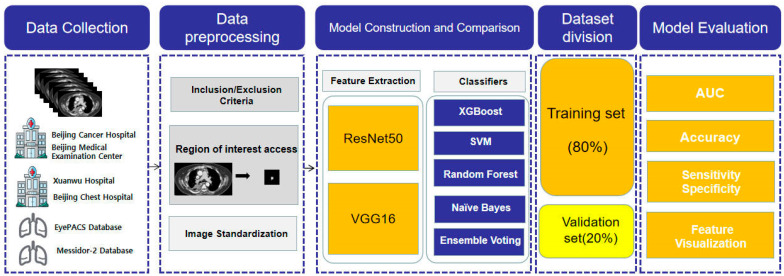
Flowchart for the design of a machine diagnostic model for benign and malignant pulmonary nodules.

In this study, data from the six aforementioned databases of patients with small pulmonary nodules were collected and acquired from finished CT scans of small pulmonary nodules using the criteria. Image preprocessing techniques such as normalization were used after initially identifying the region of interest (ROI) of lung nodules according to expert clinicians. Feature extraction was performed on the acquired CT images of the lung nodule region of interest, mostly using ResNet50 and VGG16. The nodules were then categorized as benign or malignant using five different classifiers. The dataset was divided into two parts: the training set (80%) and the validation set (20%). Finally, the model was evaluated in terms of accuracy, AUC value, specificity, and sensitivity. The specific process is shown in Figure 1.

### 2.3. Image Preprocessing

In this study, each CT image of the small lung nodule was taken as the object of this study. Semi-automatic segmentation of the whole CT image was performed by two experienced radiologists using MATLAB 2017 to segment the region of interest (ROI) using region growing method. As a result, one ROI image was obtained from each CT image. The image was also resized on the basis of the sub-base, and the resizing was set to be 32 × 32. Processing of the already intercepted images of small lung nodules was performed by means of the Adaptive Histogram Equalization (AHE) algorithm. The parameters of the AHE algorithm were set to clipLimit = 2.0 and tileGridSize = (8, 8). clipLimit controls the degree of limitation of the contrast enhancement, and tileGridSize defines the equalization region of the image. The method is based on conventional histogram equalization, where the image is divided into small blocks and histogram equalization is performed within each block to avoid introducing discontinuities between blocks. Eventually, noise reduction was performed using median filtering, which is a filtering method based on sorting statistics that uses the median value in the neighborhood around the pixel to replace the current pixel value. Median filtering is effective for removing pretzel noise or impulse noise, as well as preserving edges and details.

### 2.4. Deep Learning Algorithm

Recognizing benign and malignant lung nodules remains a popular classification job in machine vision. In general, image recognition consists of two crucial stages: picture feature extraction and feature categorization. The goal of image feature extraction is to convert the original picture data into a more expressive and identifiable feature representation. Picture characteristics can be extracted to reduce the dimensionality of picture data, eliminate extraneous information, and choose important image information. Deep learning algorithms trained on large-scale datasets extract high-level semantic characteristics from photos. ResNet50 and VGG16, two common examples of convolutional neural networks, have a significant advantage in visual feature extraction.

#### 2.4.1. ResNet50

ResNet50 addresses the vanishing gradients problem in deep neural networks, which use residual connections, allowing the network to learn residual mappings [22]. The connections avoid layers, which reduce the deterioration in deep networks. It contains 50 layers, which include convolutional, pooling, fully connected, and shortcut layers. ResNet50 is composed of a number of residual blocks with convolutional layers and shortcuts. The direct gradient flow is facilitated by the shortcut connectors [23]. The hyperparameters for extracting image features for the ResNet50 model mainly consist of two categories: weights and include_top. Weights set to ‘ImageNet’ indicates that weights pre-trained on the ImageNet dataset are used to help improve the performance and generalization of the model. Include_top set to False indicates that the top fully connected layer is not included. The hyperparameters of the ResNet50 model for extracting image features are shown specifically in Supplementary File, Table S1.

#### 2.4.2. VGG16

VGG16 is a convolutional neural network (CNN) architecture designed to build a deep network with a consistent architecture composed of repeated convolutional layers followed by max-pooling layers for spatial downsampling. By gradually increasing the depth while keeping the filter size modest (3 × 3), the network intends to learn hierarchical representations of pictures [24]. When compared to larger filters, the usage of tiny filters allows for a deeper network with fewer parameters. VGG16′s structure is distinguished by its depth, as the name suggests. It includes 16 layers, including 13 convolutional layers and 3 fully linked layers. The convolutional layers are divided into five blocks, each with several convolutional layers followed by a max-pooling layer. The completely linked layers at the network’s conclusion are in charge of categorization [25]. The parameterization of the VGG16 model is consistent with ResNet50. It is also pre-trained by ImageNet. The specific settings of VGG16 are detailed in Supplementary File, Table S2.

### 2.5. Machine Learning Classifiers

The classifiers setup is a vital task in machine learning that entails categorizing instances based on specified input data. ResNet50 and VGG16 have their own classification capabilities. However, these are frequently insufficient in categorizing finer pictures such as CT scans of lung nodules. In this work, we used the following five approaches as ResNet50 and VGG16 classifiers to build a fusion model to increase the model’s classification capabilities.

#### 2.5.1. Ensemble Voting

Ensemble Voting, a machine learning technique that integrates the predictions of numerous models to produce a final choice, was one of the methods utilized. It was founded on the idea that combining the predictions of many models might frequently result in better overall performance than using a single model alone. Ensemble Voting is widely utilized in machine learning problems like as classification and regression [26]. There are different types of voting schemes, such as majority voting, weighted voting, and soft voting. In majority voting, each model in the ensemble casts a single vote for its predicted class label, and the class label with the majority of votes is chosen as the final prediction [27]. The Ensemble Voting classifiers are composed of RandomForestClassifier, XGBClassifier, SVC (Support Vector Machine Classifier), and GaussianNB. Voting = ‘soft’ indicates the utilization of a soft voting model, implying that when classification is performed, the predictions of the base classifier are converted into probability estimates for the categories, and the best of these probabilities are voted on as the final classification result.

#### 2.5.2. Random Forest

Random Forest is based on the principle of ensemble learning, in which decision trees are trained separately on various subsets of data. Each Random Forest decision tree is built with a random selection of features and a bootstrapped sample of the original data. The final prediction is formed by collecting all of the individual tree forecasts via voting (for classification) or averaging (for regression) [28]. This randomness helps to capture different aspects of the data and improves the overall performance of the ensemble. Random Forest consists of multiple decision trees. It constructs multiple independent decision trees through random sampling and feature selection, and then produces integrated predictions by voting or averaging. Random Forest reduces overfitting, has good generalization ability, and evaluates feature importance. It is suitable for classification and regression problems and provides stable and accurate predictions. The parameter settings for the Random Forest classifier were as follows: n_estimators, 100; min_samples_leaf, 1; min_samples_split, 2; and bootstrap, True.

#### 2.5.3. XGBoost

XGBoost (eXtreme Gradient Boosting) is a powerful machine learning algorithm known for its efficiency and performance in both regression and classification tasks. XGBoost builds an ensemble of decision trees sequentially, where each tree corrects the mistakes made by the previous trees. The algorithm focuses on optimizing a specific loss function while regularizing the model to prevent overfitting [29]. In each iteration, XGBoost calculates a gradient based on the difference between the current model’s prediction and the true value, and uses this gradient to adjust the model parameters. Each new decision tree tries to correct the errors of all the previous trees and is constructed taking into account the prediction errors of the previous trees. This iterative process reduces the error and improves the predictive performance of the model gradually. The parameters of the XGBoost classifier were set as follows: binary using logisti regression; max_depth, 10; learning_rate, 0.01; and n_estimators, 100.

#### 2.5.4. SVM

Support Vector Machine (SVM) is a powerful supervised machine learning algorithm used for classification and regression tasks, which can handle both linearly separable and non-linearly separable data by using different kernel functions to transform the data into a higher-dimensional space [30]. SVM’s structure includes identifying support vectors, which are the data points closest to the decision border or hyperplane. These support vectors are critical in establishing the decision boundary and making forecasts. Depending on the situation at hand, SVM might have a linear or non-linear decision boundary, which is performed by selecting an appropriate kernel function. The SVM classifier (SVC) parameters were set as follows: strength of regularization parameters C, 1.0; break_ties, False; cache_size, 200; degree, 3; and kernel, rbf (radial basis function).

#### 2.5.5. Naïve Bayes

Naïve Bayes is a simple yet powerful machine learning algorithm based on Bayesian probability. The principle behind Nave Bayes is to utilize Bayes’ theorem to estimate the likelihood of a specific class given the observed features [31]. Given the input characteristics, it estimates the conditional probability of each class and chooses the class with the highest probability as the predicted class. The Nave Bayes structure entails creating a probabilistic model based on the training data. It calculates the prior probability of each class as well as the probability of detecting each characteristic given each class. This assumption simplifies probability computation and enables effective training and prediction. The GaussianNB parameters are set as follows: priors, None; var_smoothing, 1 × 10^−9^.

### 2.6. Feature Visualization

The collected feature information from ResNet50 and VGG16 was displayed in this study using t-SNE and feature ranking algorithms. The use of t-SNE lowered the dimensionality of the global features from 256 to 2, allowing the features to be shown on a two-dimensional scatter plot. Each data point on the plot represented a sample, and examining their spatial arrangement revealed information about the samples’ grouping, closeness, or dispersion depending on their learning attributes. This visual analysis proved useful in determining the features’ discriminative strength and separability.

### 2.7. Statistical Analysis

The statistical descriptions of patient information are presented as the mean and the standard deviation (SD) or percentage; R 4.0.3 software was used to perform the χ^2^ test or *t*-test for the basic clinical data of patients and images. The difference was statistically significant at *p* < 0.05.

Considering that deletion of observations containing missing values would result in loss of data and may affect the accuracy and reliability of subsequent analyses or modeling, the Morphological Characteristics Random Forest method was chosen to fill in the missing values. To evaluate the classification performance in the valid set, accuracy, sensitivity, specificity, positive predictive value (PPV), negative predictive value (NPV), and F1-score were calculated. In the present study, the threshold for sensitivity and specificity values was determined to be 0.5. Since we considered the current study to address the differentiation of the benign and malignant nature of lung nodules, regardless of the category to which they belonged, it is of great significance. Mean Absolute Error (MAE) is presented to evaluate the average of the distances between the model predictions and the true values of the samples. Curves from the receiver operating characteristics (ROC) were plotted to visually compare the differences between the models.

## 3. Results

### 3.1. Combined Machine Learning Models

**Figure 2:**
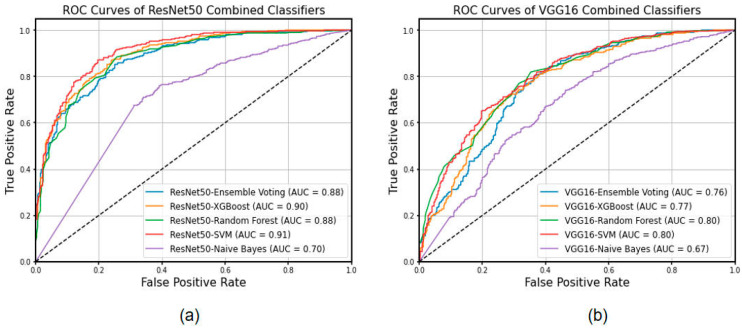
The ROC curves of different combinations of classification models in the test set. (**a**) Features extracted by ResNet50. (**b**) Features extracted by VGG16.

In this study, we utilized ResNet50 and VGG16 as the basis of feature extraction classifiers, which were federated with Ensemble Voting, XGBoost, Random Forest, SVM, and Naïve Bayes to form a new machine learning classification process. In this study, a total of 2048 features were extracted using the last convolutional layer (layer 6) of ResNet50, while a total of 512 features were extracted using the last convolutional layer (layer 5) of VGG16. The feature filtering was performed through the XGBoost process and finally 233 and 213 features were filtered in favor of the model’s classification ability, respectively. As shown in Table 3, ResNet50-Ensemble Voting achieved the best performance with an accuracy of 0.943 (0.938, 0.948) and sensitivity and specificity of 0.964 and 0.911, respectively. It was not only higher than that of the ResNet50 deep learning model, but also better than those of the comparative models with improved classifiers such as ResNet50-XGBoost. From a global perspective, the classification levels of the fusion models with the ResNet50 model as the feature extraction were significantly superior to those of VGG16. Then, the screening pass features were classified. The best AUC value (ResNet50-SVM) achieved was 0.91. In the ROC curve, each of the operating points was optimized, which also indicates the comprehensive standard of the method. The ROC curves of classification models are plotted in Figure 2.

### 3.2. Feature Visualization

**Figure 3:**
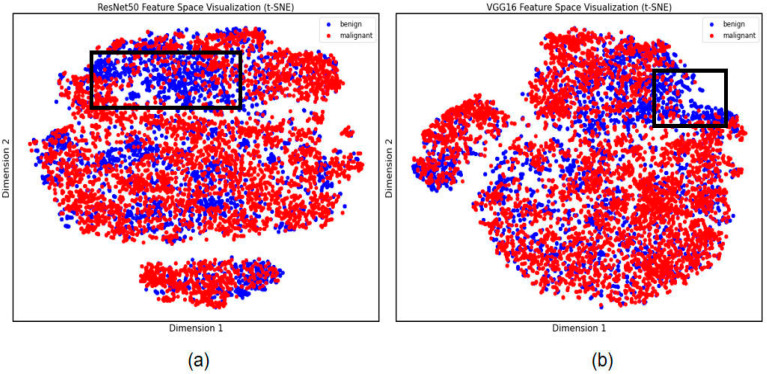
Differential feature visualization of small lung nodules. (**a**) Features extracted by ResNet50. (**b**) Features extracted by VGG16. The black box is the area of differential feature clustering.

In this study, feature filtering was performed through the XGBoost process and finally 233 and 213 features were filtered in favor of the model’s classification ability, filtered by importance of all features over 0.001. To further explore significant results in the image feature extraction results of small lung nodules, we performed further visualization of specific lung nodules. The t-SNE results demonstrate the variability in the extraction results by ResNet50 and VGG16. As shown in Figure 3, the distinction between benign and malignant lung nodules with characteristics was not discernible, but the labeled region suggested that the ResNet50 model retrieved more differentiated locales, implying that the final classification result of this model was likewise superior.

**Figure 4:**
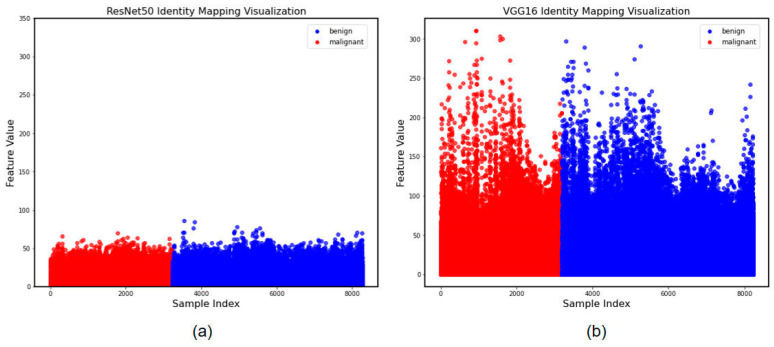
Identity Mapping of visualization of the effectiveness of the learned features. (**a**) Features extracted by ResNet50. (**b**) Features extracted by VGG16.

To further demonstrate the feature extraction differences between the two methods, we categorized and presented the differential feature locations using the Identity Mapping method. Through Figure 4, we discovered that there was little differentiation between the two methods of extracting features as a whole; however, for small malignant lung nodules, ResNet50 discovered more diversified features, demonstrating the efficiency of the feature extraction strategy. However, the less relevant particular aspects were considered.

**Figure 5:**
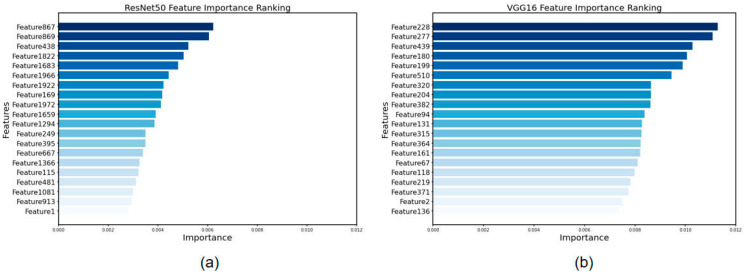
Feature importance ranking for feature screening via XGBoost. (**a**) Features extracted by ResNet50 and (**b**) features extracted by VGG16.

Based on our findings, we ordered the features from most important to least important and selected the top 20 most important features recovered by the ResNet50 and VGG16 algorithms. Among the ResNet50 results, Feature 867, Feature 869, and Feature 438 were determined to be the most important. In the instance of VGG16, Feature 228, Feature 277, and Feature 439 were selected as the most essential characteristics, in that order. The results are shown in Figure 5.

## 4. Discussion

Accurate evaluation of the benign and malignant nature of small lung nodules (<20 mm) detected in CT is essential for the early diagnosis and management of lung cancer, and it has remained a challenging undertaking during clinical practice [32]. In this study, we developed and validated a classification diagnostic model combining deep learning and machine learning to distinguish between benign and malignant early lung nodules using CT images of small lung nodules from six different databases. Our results demonstrated that our proposed method, ResNet50-Ensemble Voting, achieved superior performance, reaching an accuracy of 0.943 (0.938, 0.948) along with a sensitivity of 0.964 and specificity of 0.911. In addition, ResNet50-SVM achieved an AUC of 0.91, and the accuracy attained 0.83 (0.82, 0.85). In the ROC curve, each of the operating points was optimized, which also indicates the comprehensive standard of the method. This result showed the competence of diagnosing the benign and malignant nature of small lung nodules in the validation set. This study further demonstrated the feature extraction capability of ResNet50 and VGG16, visualized the features, and compared the performance of the combined model in diagnosing the benign and malignant nature of lung nodules. The early detection and identification of lung nodules are particularly critical and challenging, especially when the gold standard of pathological tissue is not available. In this context, the ResNet50-SVM and ResNet50-XGBoost models developed in this study made significant contributions by selecting the best combination of feature extraction and classifiers. This could improve the diagnostic capabilities for small lung nodules and reduce the misdiagnosis and missed diagnosis rates among clinicians. Ultimately, it provides clearer diagnostic guidance for patients in the early stages of lung cancer.

In recent years, ResNet50 and VGG16 have been applied as the most classical CNN network models for diagnosis and recognition of diseases [22,33,34]. ResNet50 has a deeper network depth compared to traditional deep networks to better capture details and semantic information in images [35]. VGG16, on the other hand, is able to capture features at different scales by stacking multiple small convolutional kernels and pooling layers to increase the nonlinear expressiveness of the network [36]. Both methods have demonstrated competence in the diagnosis of the nature of pulmonary nodules.

There are numerous studies that have utilized residual networks to classify lung cancer. One study excluded the results of a multilevel crossover residual network for lung nodule classification, which could reach an 85.88% accuracy rate [37]. Zhang used ResNet as the basic framework combined with CBAM to classify conventional lung nodules, and the AUC could reach more than 0.95 [38]. Xie utilized the collaborative deep learning of knowledge in a staging chest CT of benign and malignant lung nodules with an accuracy of up to 95.70% [39]. Wang built a multi-scale residual network (MResNet) to accurately extract the features of lung nodules and classified them in conjunction with deep learning, achieving an accuracy of 99.12% [40]. This shows that the research on regular lung nodules is well established, but the diagnosis of small, early lung nodules needs to be further clarified. In addition, consideration and improvements should be made to the related research methods.

The current study focused more on the nature of small lung nodules in the early stages of lung cancer. Size and growth are crucial factors in evaluating the malignant potential of a nodule. The likelihood of malignancy is positively correlated with nodule diameter, and therefore the importance of morphology in CT images should not be underestimated [41]. Farjah primarily discovered the relationship between lung cancer diagnosis and nodal features using multivariate analysis, and the created model had an AUC of 0.75, indicating that detection capacity needed to be improved further [42]. Wookjin classified the early imaging features of lung cancer by low-dose CT lung nodules with an AUC value of 0.89. Although this study targeted nodules in the early stages of lung cancer, it did not account for the specific size of the nodules [43]. DNA promoter hypermethylation was found to be diagnostic for early-stage lung cancer, and specific markers such as SOX17, TAC1, and HOXA7 were shown to be diagnostic at an AUC of 0.89 [44]. Tumor necrosis factor-α receptor-associated protein (TRAP1) was also of significance in the diagnostic process of lung nodules in the early stages of lung cancer, with an AUC value of approximately 0.835 [45]. Relevant biomarkers, despite displaying good performance, prevented screening from being applied to broad populations due to their expensive cost. On this premise, the current findings enhanced the diagnosis of early lung nodules. This study additionally demonstrated the characteristics from various viewpoints and attempted to investigate the capacity of various aspects to contribute. Not only is our proposed method noninvasive, but its cost is also readily acceptable compared to biomarkers.

Nonetheless, our study had several drawbacks. First and foremost, because this was a study of small lung nodules, the gold standard could not be achieved. The aim was just to bring the method as close to the physician’s diagnostic level as possible. Second, the model was not combined and compared with radiologic features. Despite the fact that both were related to imaging, the method proposed in this study cannot directly provide information such as clinical indications such as the burr sign. Finally, one shortcoming of the technique was that it required a high number of precisely labeled counts, making data collection a greater challenge.

## 5. Conclusions

In conclusion, the combined machine learning model ResNet50-Ensemble Voting showed remarkable performance in the identification of benign and malignant small pulmonary nodules (<20 mm) from multiple centers. The combined feature visualization process further clarifies the variability in different features. The model can help clinicians accurately diagnose the nature of early-stage lung sub-nodules in clinical practice.

## Figures and Tables

**Table 1:** The checklist of subjects and images.

Database	Subjects (*n*, %)	Images (*n*, %)
Beijing Chest Hospital	43 (10.86)	89 (10.67)
Beijing Cancer Hospital	106 (26.77)	228 (27.37)
Xuanwu Hospital	96 (24.24)	204 (24.46)
Beijing Physical Examination Center	79 (19.95)	175 (20.98)
TCGA Public Database	26 (6.57)	51 (6.12)
LIDC-IDRI	46 (11.62)	87 (10.44)
Total	396 (100.00)	834 (100.00)

**Table 2:** Comparison of clinical information between benign group and malignant group.

Clinic Information	Benign (*n* = 154)	Malignant (*n* = 242)	*p*
Age (years, mean ± SD)	61.43 ± 12.38	68.42 ± 10.29	0.057 ^a^
Gender (*n*, %)			0.903 ^b^
Male	81 (0.53)	135 (0.56)	
Female	73 (0.47)	107 (0.44)	

^a^ *t*-test was used for the distribution difference of continuous variables. ^b^ Chi-square test was used for the distribution difference of categorical variables.

**Table 3:** Classification and diagnosis of diabetic nephropathy based on the migration model.

Models	Accuracy	Sensitivity	Specificity	PPV	NPV	AUC	MAE	F1-Score
ResNet50	0.75 (0.73, 0.77)	0.82	0.66	0.78	0.71	0.81	0.27	0.80
VGG16	0.61 (0.59, 0.63)	0.37	0.90	0.82	0.54	0.61	0.40	0.51
VGG16-Ensemble Voting	0.88 (0.88, 0.89)	0.95	0.78	0.74	0.54	0.77	0.11	0.91
VGG16-XGBoost	0.74 (0.72, 0.77)	0.86	0.57	0.73	0.68	0.76	0.25	0.80
VGG16-Random Forest	0.73 (0.71, 0.75)	0.89	0.49	0.72	0.74	0.79	0.27	0.80
VGG16-SVM	0.72 (0.70, 0.75)	0.90	0.46	0.72	0.76	0.78	0.27	0.80
VGG16-Naïve Bayes	0.63 (0.61, 0.66)	0.69	0.54	0.69	0.55	0.66	0.37	0.69
ResNet50-Ensemble Voting	0.94 (0.93, 0.94)	0.96	0.91	0.85	0.63	0.88	0.06	0.95
ResNet50-XGBoost	0.82 (0.80, 0.83)	0.89	0.70	0.82	0.81	0.90	0.18	0.86
ResNet50-Random Forest	0.82 (0.80, 0.84)	0.92	0.66	0.81	0.86	0.89	0.19	0.86
ResNet50-SVM	0.83 (0.82, 0.85)	0.93	0.69	0.82	0.86	0.91	0.17	0.87
ResNet50-Naïve Bayes	0.71 (0.69, 0.73)	0.75	0.66	0.77	0.63	0.75	0.29	0.76

## Data Availability

The data presented in this study are available in this article (and Supplementary Materials).

## Supplementary Materials

The following supporting information can be downloaded at: https://www.mdpi.com/article/10.3390/cancers15225417/s1, Table S1: The ResNet50 model was pre-trained with ImageNet to extract hyper-parameter information of image features; Table S2: The VGG16 model was pre-trained with ImageNet to extract hyper-parameter information of image features.
