# Supplementary material for: Machine Learning Model of ResNet50-Ensemble Voting for Malignant–Benign Small Pulmonary Nodule Classification on Computed Tomography Images
Source: Cancers (Basel). 2023 Nov 15;15(22):5417. doi: 10.3390/cancers15225417 (PMC10670717; doi:10.3390/cancers15225417)
Supplement: Supplementary file 1 [file cancers-15-05417-s001.zip › cancers-2608954-supplementary.pdf]

Supplementary Materials

Table S1: The ResNet50 model was pre-trained with ImageNet to extract hyper-parameter information of image features.

Layer Name	Weighting Shapes
input_3	-
conv1_pad	-
conv1_conv	(7, 7, 3, 64)
conv1_bn	(64,)
conv1_relu	-
pool1_pad	-
pool1_pool	-
conv2_block1_1_conv	(1, 1, 64, 64)
conv2_block1_1_bn	(64,)
conv2_block1_1_relu	-
conv2_block1_2_conv	(3, 3, 64, 64)
conv2_block1_2_bn	(64,)
conv2_block1_2_relu	-
conv2_block1_0_conv	(1, 1, 64, 256)
conv2_block1_3_conv	(1, 1, 64, 256)
conv2_block1_0_bn	(256,)
conv2_block1_3_bn	(256,)
conv2_block1_add	-
conv2_block1_out	-
conv2_block2_1_conv	(1, 1, 256, 64)
conv2_block2_1_bn	(64,)
conv2_block2_1_relu	-
conv2_block2_2_conv	(3, 3, 64, 64)
conv2_block2_2_bn	(64,)
conv2_block2_2_relu	-
conv2_block2_3_conv	(1, 1, 64, 256)
conv2_block2_3_bn	(256,)
conv2_block2_add	-
conv2_block2_out	-
conv2_block3_1_conv	(1, 1, 256, 64)
conv2_block3_1_bn	(64,)
conv2_block3_1_relu	-
conv2_block3_2_conv	(3, 3, 64, 64)
conv2_block3_2_bn	(64,)
conv2_block3_2_relu	-
conv2_block3_3_conv	(1, 1, 64, 256)
conv2_block3_3_bn	(256,)
conv2_block3_add	-
conv2_block3_out	-

conv3_block1_1_conv	(1, 1, 256, 128)
conv3_block1_1_bn	(128,)
conv3_block1_1_relu	-
conv3_block1_2_conv	(3, 3, 128, 128)
conv3_block1_2_bn	(128,)
conv3_block1_2_relu	-
conv3_block1_0_conv	(1, 1, 256, 512)
conv3_block1_3_conv	(1, 1, 128, 512)
conv3_block1_0_bn	(512,)
conv3_block1_3_bn	(512,)
conv3_block1_add	-
conv3_block1_out	-
conv3_block2_1_conv	(1, 1, 512, 128)
conv3_block2_1_bn	(128,)
conv3_block2_1_relu	-
conv3_block2_2_conv	(3, 3, 128, 128)
conv3_block2_2_bn	(128,)
conv3_block2_2_relu	-
conv3_block2_3_conv	(1, 1, 128, 512)
conv3_block2_3_bn	(512,)
conv3_block2_add	-
conv3_block2_out	-
conv3_block3_1_conv	(1, 1, 512, 128)
conv3_block3_1_bn	(128,)
conv3_block3_1_relu	-
conv3_block3_2_conv	(3, 3, 128, 128)
conv3_block3_2_bn	(128,)
conv3_block3_2_relu	-
conv3_block3_3_conv	(1, 1, 128, 512)
conv3_block3_3_bn	(512,)
conv3_block3_add	-
conv3_block3_out	-
conv3_block4_1_conv	(1, 1, 512, 128)
conv3_block4_1_bn	(128,)
conv3_block4_1_relu	-
conv3_block4_2_conv	(3, 3, 128, 128)
conv3_block4_2_bn	(128,)
conv3_block4_2_relu	-
conv3_block4_3_conv	(1, 1, 128, 512)
conv3_block4_3_bn	(512,)
conv3_block4_add	-
conv3_block4_out	-
conv4_block1_1_conv	(1, 1, 512, 256)
conv4_block1_1_bn	(256,)

conv4_block1_1_relu	-
conv4_block1_2_conv	(3, 3, 256, 256)
conv4_block1_2_bn	(256,)
conv4_block1_2_relu	-
conv4_block1_0_conv	(1, 1, 512, 1024)
conv4_block1_3_conv	(1, 1, 256, 1024)
conv4_block1_0_bn	(1024,)
conv4_block1_3_bn	(1024,)
conv4_block1_add	-
conv4_block1_out	-
conv4_block2_1_conv	(1, 1, 1024, 256)
conv4_block2_1_bn	(256,)
conv4_block2_1_relu	-
conv4_block2_2_conv	(3, 3, 256, 256)
conv4_block2_2_bn	(256,)
conv4_block2_2_relu	-
conv4_block2_3_conv	(1, 1, 256, 1024)
conv4_block2_3_bn	(1024,)
conv4_block2_add	-
conv4_block2_out	-
conv4_block3_1_conv	(1, 1, 1024, 256)
conv4_block3_1_bn	(256,)
conv4_block3_1_relu	-
conv4_block3_2_conv	(3, 3, 256, 256)
conv4_block3_2_bn	(256,)
conv4_block3_2_relu	-
conv4_block3_3_conv	(1, 1, 256, 1024)
conv4_block3_3_bn	(1024,)
conv4_block3_add	-
conv4_block3_out	-
conv4_block4_1_conv	(1, 1, 1024, 256)
conv4_block4_1_bn	(256,)
conv4_block4_1_relu	-
conv4_block4_2_conv	(3, 3, 256, 256)
conv4_block4_2_bn	(256,)
conv4_block4_2_relu	-
conv4_block4_3_conv	(1, 1, 256, 1024)
conv4_block4_3_bn	(1024,)
conv4_block4_add	-
conv4_block4_out	-
conv4_block5_1_conv	(1, 1, 1024, 256)
conv4_block5_1_bn	(256,)
conv4_block5_1_relu	-
conv4_block5_2_conv	(3, 3, 256, 256)

conv4_block5_2_bn	(256,)
conv4_block5_2_relu	-
conv4_block5_3_conv	(1, 1, 256, 1024)
conv4_block5_3_bn	(1024,)
conv4_block5_add	-
conv4_block5_out	-
conv4_block6_1_conv	(1, 1, 1024, 256)
conv4_block6_1_bn	(256,)
conv4_block6_1_relu	-
conv4_block6_2_conv	(3, 3, 256, 256)
conv4_block6_2_bn	(256,)
conv4_block6_2_relu	-
conv4_block6_3_conv	(1, 1, 256, 1024)
conv4_block6_3_bn	(1024,)
conv4_block6_add	-
conv4_block6_out	-
conv5_block1_1_conv	(1, 1, 1024, 512)
conv5_block1_1_bn	(512,)
conv5_block1_1_relu	-
conv5_block1_2_conv	(3, 3, 512, 512)
conv5_block1_2_bn	(512,)
conv5_block1_2_relu	-
conv5_block1_0_conv	(1, 1, 1024, 2048)
conv5_block1_3_conv	(1, 1, 512, 2048)
conv5_block1_0_bn	(2048,)
conv5_block1_3_bn	(2048,)
conv5_block1_add	-
conv5_block1_out	-
conv5_block2_1_conv	(1, 1, 2048, 512)
conv5_block2_1_bn	(512,)
conv5_block2_1_relu	-
conv5_block2_2_conv	(3, 3, 512, 512)
conv5_block2_2_bn	(512,)
conv5_block2_2_relu	-
conv5_block2_3_conv	(1, 1, 512, 2048)
conv5_block2_3_bn	(2048,)
conv5_block2_add	-
conv5_block2_out	-
conv5_block3_1_conv	(1, 1, 2048, 512)
conv5_block3_1_bn	(512,)
conv5_block3_1_relu	-
conv5_block3_2_conv	(3, 3, 512, 512)
conv5_block3_2_bn	(512,)
conv5_block3_2_relu	-

conv5_block3_3_conv	(1, 1, 512, 2048)
conv5_block3_3_bn	(2048,)
conv5_block3_add	-
conv5_block3_out	-

Table S2: The VGG16 model was pre-trained with ImageNet to extract hyper-parameter information of image features.

Layer Name	Weighting Shapes
input_4	-
block1_conv1	[(3, 3, 3, 64), (64,)]
block1_conv2	[(3, 3, 64, 64), (64,)]
block1_pool	-
block2_conv1	[(3, 3, 64, 128), (128,)]
block2_conv2	[(3, 3, 128, 128), (128,)]
block2_pool	-
block3_conv1	[(3, 3, 128, 256), (256,)]
block3_conv2	[(3, 3, 256, 256), (256,)]
block3_conv3	[(3, 3, 256, 256), (256,)]
block3_pool	-
block4_conv1	[(3, 3, 256, 512), (512,)]
block4_conv2	[(3, 3, 512, 512), (512,)]
block4_conv3	[(3, 3, 512, 512), (512,)]
block4_pool	-
block5_conv1	[(3, 3, 512, 512), (512,)]
block5_conv2	[(3, 3, 512, 512), (512,)]
block5_conv3	[(3, 3, 512, 512), (512,)]
block5_pool	-
